# Recent advances and challenges in the interaction between myofibrillar proteins and flavor substances

**DOI:** 10.3389/fnut.2024.1378884

**Published:** 2024-04-25

**Authors:** Rong Qian, Chang Sun, Ting Bai, Jing Yan, Jie Cheng, Jiamin Zhang

**Affiliations:** ^1^College of Food and Biological Engineering, Chengdu University, Chengdu, China; ^2^Meat Processing Key Laboratory of Sichuan Province, Chengdu, China; ^3^Sichuan Laochuan East Food Co., Ltd., Chengdu, China

**Keywords:** myofibrillar protein, flavor substance, binding interaction, volatile flavor, influencing factor

## Abstract

Myofibrillar proteins are an important component of proteins. Flavor characteristics are the key attributes of food quality. The ability of proteins to bind flavor is one of their most fundamental functional properties. The dynamic balance of release and retention of volatile flavor compounds in protein-containing systems largely affects the sensory quality and consumer acceptability of foods. At present, research on flavor mainly focuses on the formation mechanism of flavor components, while there are few reports on the release and perception of flavor components. This review introduces the composition and structure of myofibrillar proteins, the classification of flavor substances, the physical binding and chemical adsorption of myofibrillar proteins and volatile flavor substances, as well as clarifies the regulation law of flavor substances from the viewpoint of endogenous flavor characteristics and exogenous environment factors, to provide a theoretical reference for the flavor regulation of meat products.

## 1 Introduction

Myofibrillar protein (MP) is a group of salt-soluble structural proteins with biological functions, and it is also an important component of contracting muscle fibers in muscle (1). However, MP itself does not have odor, but it can interact with flavor compounds through specific molecular bonds to enhance and regulate the overall flavor of meat products, thus affecting the flavor intensity of food (2). MP can interact with flavor compounds through special molecular bonds, which can affect the flavor intensity of the food (3). Through physical or chemical interaction with flavor compounds, MP can adsorb volatile flavor substances, affect the flavor intensity of food to different degrees, change the overall balance of flavor, and thus affect the release of flavor (4).

Because sensory organs are very sensitive to changes in flavor levels, interactions between flavor compounds and other components in food can have a profound effect on flavor perception by altering the rate at which volatile flavors are released. Protein in meat products provides a complex scientific site for interaction with flavor compounds, protein molecules can bind and separate lipophilic flavor molecules, so that adverse flavor in food is transmitted to food, seriously affecting the sensory and edible quality of food (5). Graf et al. (6) first observed the transmission of this odor property in some products containing whey protein and soy protein. The flavor adsorption properties of proteins are largely affected by the conformational state of proteins and the factors that change the conformational state (7). In addition, it is also affected by exogenous factors such as temperature and pH, which ultimately lead to changes in food flavor and quality.

Flavor is one of the important factors for consumers to choose meat products (8). The adsorption and release of protein and flavor substances will affect the sensory experience of meat products to some extent, so that adverse flavor in food is transmitted to food, which seriously affects the sensory and edible quality of food. There are reversible non-covalent bonds and irreversible covalent bonds in the binding process of protein-aroma compounds. Covalent bond cooperation mainly includes covalent cross-linking of aldehyde groups (–CHO), carbonyl groups (C = O) and amino acid residues (–NH_2_ and –SH) (9). Studies have shown that with the increase of trypsin content, the adsorption capacity of myosin to aldehydes and ketones is enhanced, mainly due to the increase of amino activity and sulfhydryl group content, which leads to the development of the secondary structure of proteins, thus enhancing the binding behavior of proteins with volatile components (10). In addition, the ε-amino group of the lysine residue side chain also binds with aldehydes through the formation of Schiff bases. Anantharamkrishnan et al. (11) found that milk proteins can be covalently bonded with aldehydes, mercaptans and furans through Schiff bases, the formation of disulfide bonds and the Michael addition reaction. In addition, some amine volatiles form amide bonds with the terminal carboxyl groups of aspartate and glutamate, which also belong to covalent bonding (12). The formation rate of these combinations is slow or fast, which can lead to changes in food flavor characteristics.

There are many definitions of food flavor, which was first proposed by Hall R. L. in 1986. He believes that food flavor is the sum of sensory impressions produced by people’s sensory organs after ingesting oral food, including smell, taste, touch, temperature and pain (13). There are thousands of known flavor substances, which can be divided into hydrocarbons, aldehydes, ketones, alcohols, esters, sulfur compounds, furans, pyrazines and so on (14). Protein degradation can promote the formation of flavor substances, and it can change the headspace concentration through adsorption of volatile components by molecular bonds (15), it also affects the gel and texture of food. By increasing the mass transfer resistance of volatile components, the overall flavor balance can be changed to different degrees, which has a great impact on the flavor release of food.

## 2 Composition

**Figure 1:**
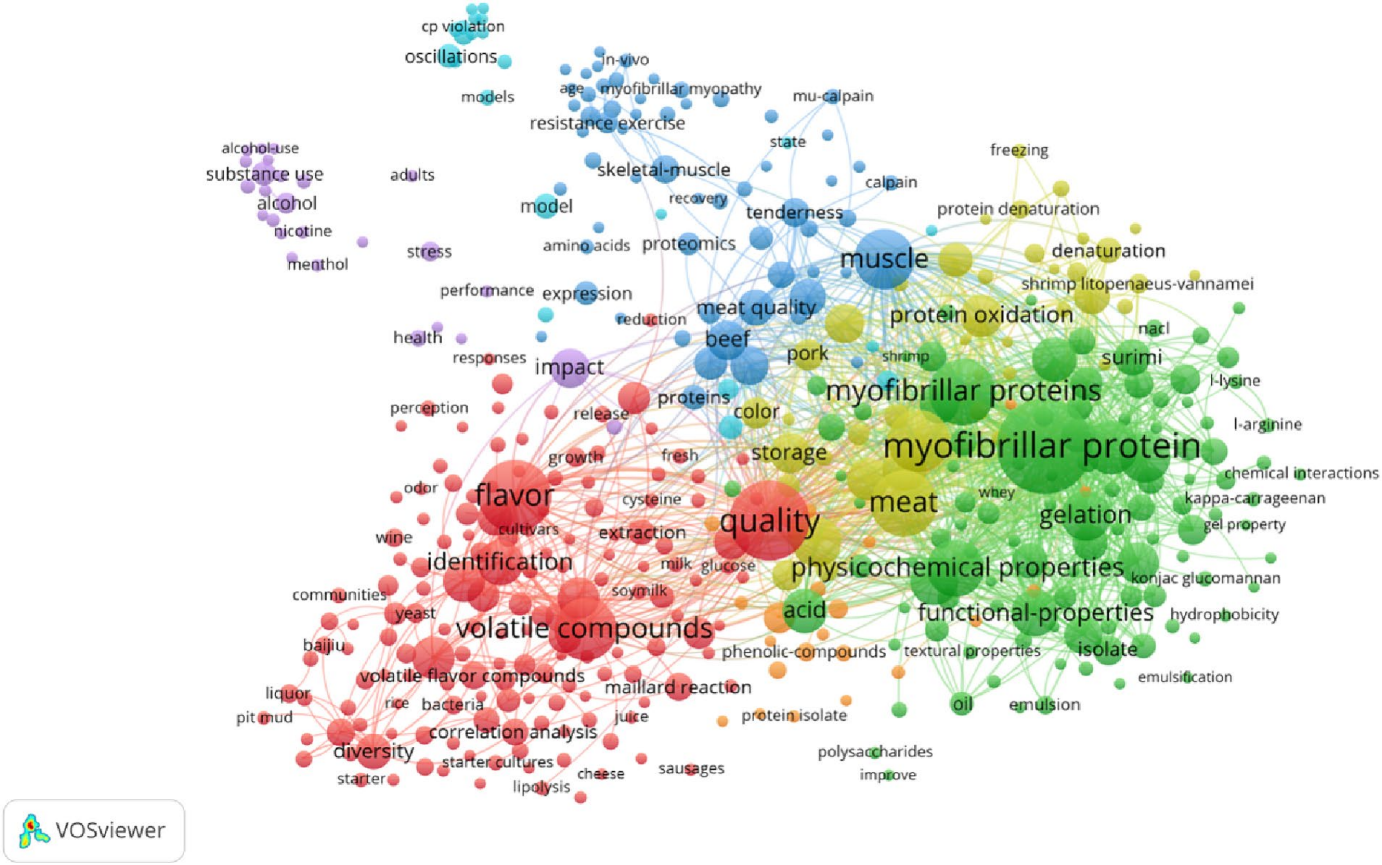
Sankey diagram of MP and flavor substances.

This review mainly introduces the composition and structure of MP, the classification of flavor substances, the physical binding and chemisorption of MP and volatile flavor substances, and the influencing factors (see Figure 1).

### 2.1 The composition of myofibrillar protein

**Figure 2:**
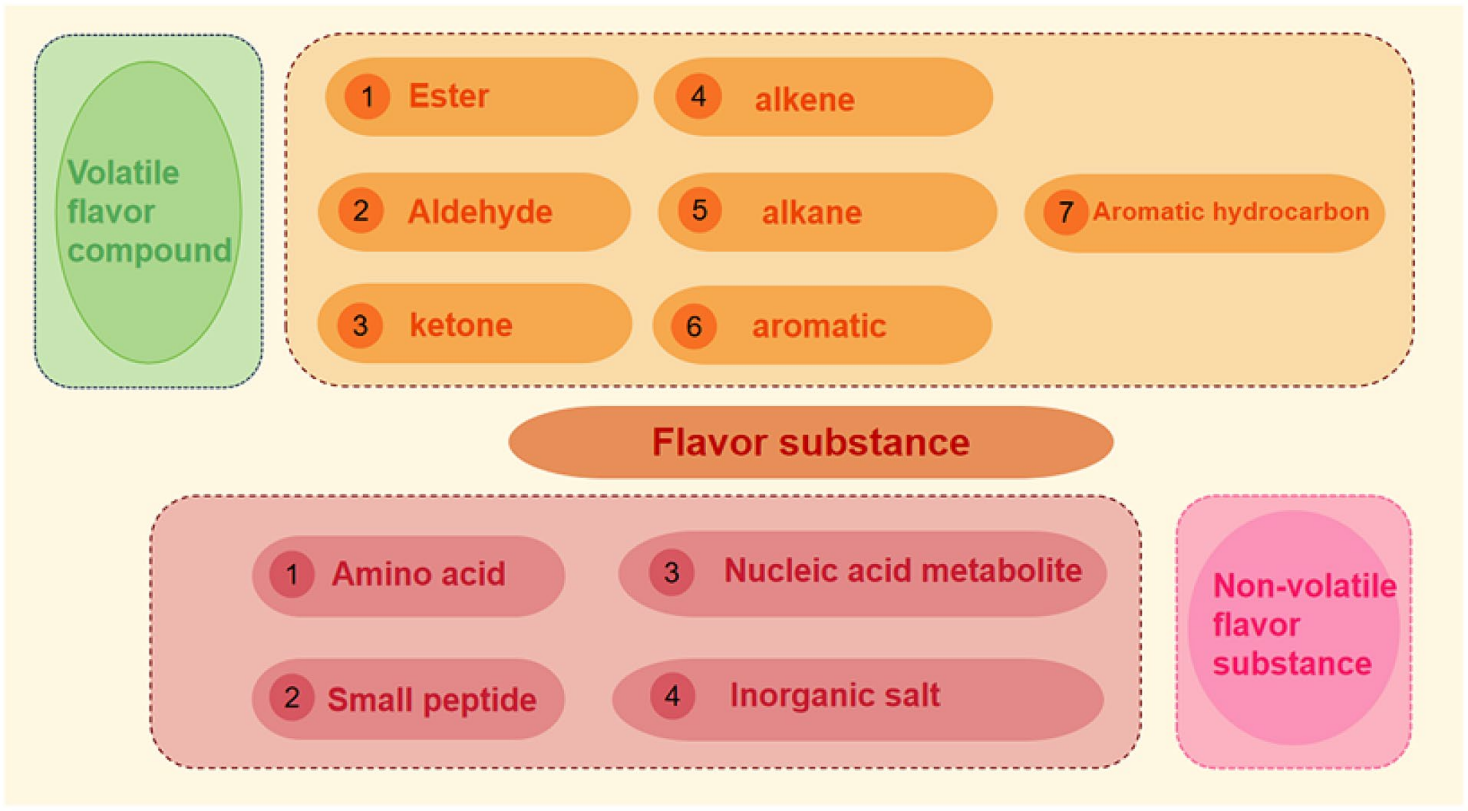
Classification of flavor substances.

MP is divided into three categories according to their composition structure: (1) filament in, which is mainly composed of myosin and actin (2) regulatory proteins, including the tropomyosin-troponin complex, α-/β-actinin, M-protein and C-protein (3) scaffold protein, which consists of titin, associated actin (nebulin), desmin, vimentin and synemin (16), myosin and actin participate in the contraction process of muscles and combine to form actomyosin. The adsorption capacity of G-actin and myosin in MP to volatile flavor substances is different. Pérez-Juan et al. (15) find that G-actin and myosin in dry-cured ham were mainly bound to flavor components with small partition coefficients. Cao et al. (17) found that different concentrations of H_2_O_2_ affected the changes of α dynamic helix, carbonyl group, hydrogen bond, sulfhydryl group and hydrophobic stage point in G-actin, thus causing differences in its adsorption capacity for alcohols and aldehydes (Figure 2).

### 2.2 Classification of flavor substances

**Figure 3:**
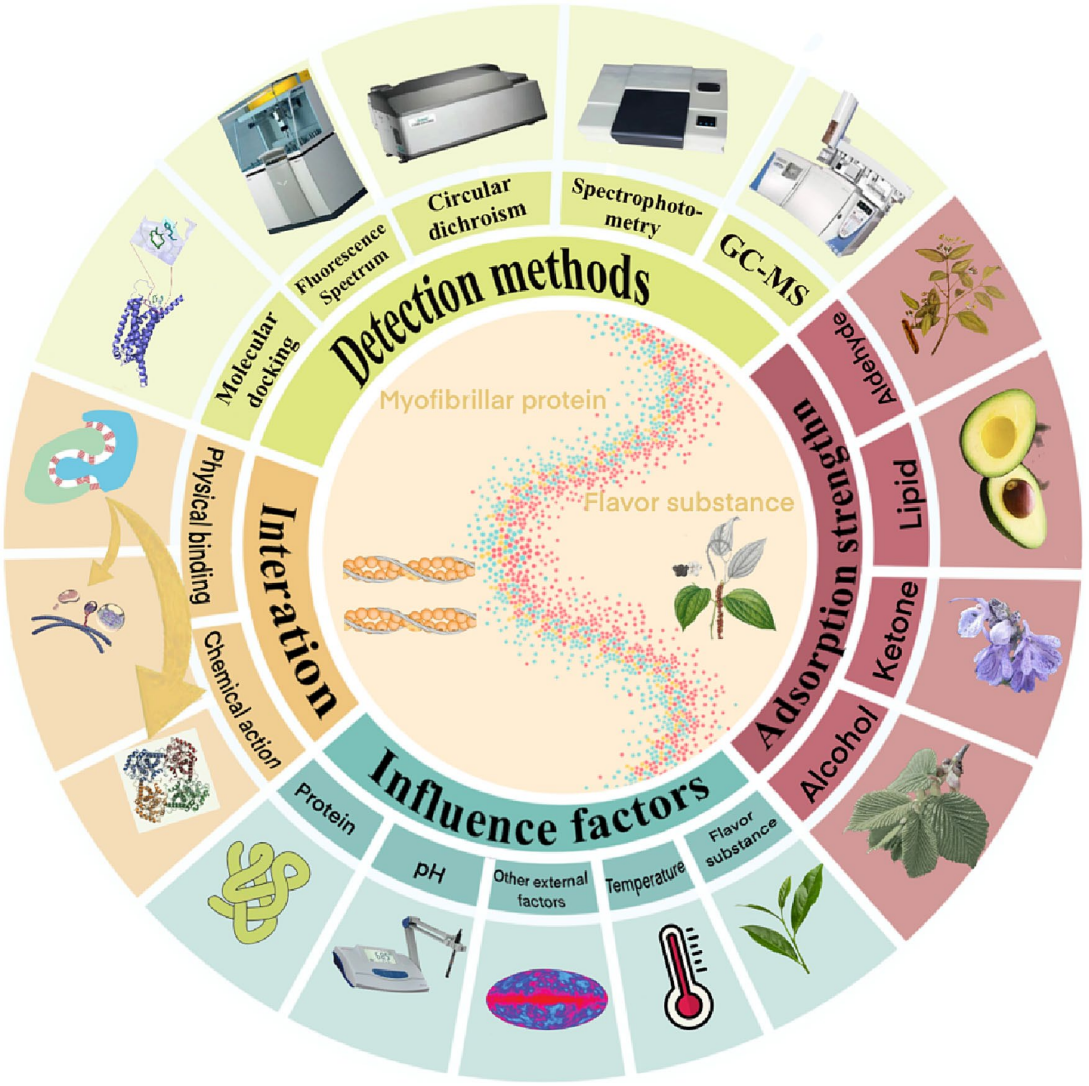
The interaction between MP and flavor substance and its influencing factors and detection methods.

Flavor not only includes aroma, but also taste. At present, a variety of flavor substances produced in meat products can be roughly divided into two categories: one is volatile flavor substances, whose production and existence of flavor substances can be released from food into the environment, causing changes in food flavor profile. The other is non-volatile flavor substances, such as inorganic salts, free amino acids, small peptides and nucleic acid metabolites such as inosine acid, ribose and so on. Flavor precursors themselves do not produce flavor, and the products after the reaction of flavor precursors are usually summarized as odor and taste (18). The research on odor mainly focuses on volatile components, while the research on taste mostly focuses on non-volatile components (see Figure 3).

#### 2.2.1 Volatile flavor compounds

The most important contribution to the characteristic flavor of meat products is the volatile flavor substance (19). Volatile flavor substances can be divided into esters, aldehydes, ketones, alkanes, aromatics, alkenes and halohydrocarbons (20). Muscle protein itself has no fragrance, and processed meat products will produce specific flavor compounds. In different processing methods, due to different heat transfer methods, meat products will produce and release a large number of flavor compounds through fat oxidation and degradation. Maillard reaction and degradation of flavor precursor substances, such as: Alcohol, aldehydes, carboxylic acid, ester, furan, pyridine, pyrazine, thiazole, thiophene, nitrogen, sulfur, etc., and then produce meat flavor (21). Song et al. (22) reported that one-hundred-and-nine volatile compounds were identified by GC-MS using SPME and SDE methods in two braised porks. From the SDE-AEDA-GC/O analysis, it was found that pentanal (almond, pungent), nonanal (fat, green) (E, E)-2,4-decadienal (fat, roast), phenyl acetaldehyde (hawthorne, honey, sweet), dodecanal (lily, fat, citrus) and linalool showed the highest OAV values (>200), indicating a contribution to the aroma of braised pork.

#### 2.2.2 Non-volatile flavor substance

Non-volatile flavor substances, namely non-volatile flavor substances, determine the flavor characteristics of meat products. Flavor compounds refer to non-volatile or water-soluble substances that have taste or sense of touch, and are often the precursor substances of volatile flavor substances. The taste substances in meat products mainly include amino acids, small peptides, nucleic acid metabolites and inorganic salts, etc. (18). Chiang et al. (23) studied the non-volatile flavor substances in chicken soup, pork soup, mushroom soup and seafood soup, and the analysis showed that guanylic acid, inosine acid and xanthylate played the main role in flavor in the broth.

## 3 Interaction of myofibrillar protein with flavor substance

The original and processed flavor substances of meat and meat products mainly include sulfur compounds, oxygen-containing heterocyclic compounds, nitrogen-containing heterocyclic compounds, aldehydes, ketones, alcohols, acids and lactones (24). The interaction of proteins with flavor substances mainly consists of physical binding and chemical interactions. In meat and meat products, hydrophobic interaction is the main force to maintain the interaction between protein and flavor substances (7). The use of reversible combinations can be used to reduce flavor loss during processing and re-release of flavor components during consumption, while irreversible combinations are important for the removal of off-flavors from foods. Anantharamkrishnan and Reineccius (25) used Fourier infrared spectroscopy, nuclear magnetic resonance, equilibrium dialysis and other analytical methods to study the flavor combination sites of external lactoglobulin, and confirmed that the flavor binding sites of globulin-lactoglobulin were mostly distributed in the protein hydrophobic region, and there were multiple different combination sites and secondary combination sites with weak effects. Various flavor components either bind to the hydrophobic region on the outer surface of the protein, or act on the central hole of the protein. However, which flavor components each of these action sites tends to bind to, and the specific effects on the conformation of the protein, are unclear. The binding mechanism of protein and volatile flavor components still needs to be further studied.

### 3.1 Adsorption mechanism of myofibrillar protein and volatile flavor substances

The diversity of amino acid side chain structure enables proteins to interact with volatile flavor components in a variety of forces, most of which are reversible, including ionic bonds, hydrogen bonds and hydrophobic interactions (26). The covalent bond between the two is irreversible, mainly including the covalent cross-linking of aldehydes and the amino group of the side chain of lysine residues through the condensation of aldehyde group and imino to Schiff base and the binding of sulfur compounds to proteins (4) (Table 1). In addition, proteins can physically adsorb volatile components through van der Waals force and capillary adsorption.

**Table 1:** Interaction mechanism and main influencing factors between myofibrillar protein and flavor substances.

Protein	Flavor substance	Adsorption mechanism	Main influence factors	References
Myofibrillar protein	Volatile flavor compounds [maxim, essential oil (ZBMEO), malondialdehyde]	The addition of volatile flavor substances can reduce the surface hydrophobicity index and particle size of MP, enhance the stability of protein structure	Types of volatile flavor substances	(27–29)
Myofibrillar protein	Non-volatile flavor; free amino acids	The breakdown of flavor compounds leads to a reduction in non-flavor substances	Heat treatment temperature, time, cold storage time	(30, 31)
Myofibrillar protein	Polyphenol [chlorogenic acid (CGA); quercetin (QUE)]	The binding of CGA to MP depends on van der Waals forces and hydrogen bonds, while the binding of QUE to MP is based on electrostatic interactions	Detection method	(32, 33)
Myosin	Aldehyde, ketone	The interactions between proteins and aldehydes and ketones include reversible hydrogen bond, hydrophobic bond, ionic bond, van der Waals force and irreversible covalent bond	Proteolysis, heat treatment, protein conformation	(34, 35)
Myosin	Low molecular weight additives (metal ions, phosphates, amino acids, hydrolysates, phenols and edible oils, myristin, anisole, artemisol)	Low molecular weight additive types affect covalent, ionic and hydrogen bonds and enhance hydrophobic interactions	Types of low molecular additives, ultrasound	(36, 37)

#### 3.1.1 Physical binding

The physical combination between proteins and flavor substances is mainly achieved by van der Waals forces and electrostatic forces, including van der Waals forces and capillary adsorption, which are reversible. In general, flavor compounds are physically trapped in the capillaries and fissures of proteins to influence their flavor properties, and non-polar compounds such as hydrocarbons, lipids, and proteins have reversible hydrophobic interactions. Polar compounds such as alcohols interact with proteins to form hydrogen bonds, and fatty acids generally interact with proteins in an electrostatic manner (38).

#### 3.1.2 Chemical action

Chemical interactions include reversible weak hydrophobic interactions, strong ionic effects, and irreversible strong covalent bonds, namely electrostatic adsorption, hydrogen bonding, and covalent bonding (39). Some protein groups can have strong binding with some flavor substances, such as aldehydes and ketones volatiles can form a Schiff base with the terminal amino group of lysine, while some amine volatiles can form an amide bond with the terminal carboxyl group of aspartate and glutamate, and aldehyde group compounds can form a Schiff base with amino acids to covalently bind proteins (40).

Some flavor molecules can interact with the side chains of proteins via covalent bonds, including aldehyde-lysine and amine-carbonyl. These interactions are usually irreversible. The binding of sulfur compounds to proteins can also be classified as covalent interactions (9, 41). Anantharamkrishnan et al. (11) showed that covalent bonds were formed between β-lactoglobulin and aldehydes, mercaptans and furans containing functional groups, resulting in Schiff base, Michael addition and disulfide bonds. The formation of covalent bonds between proteins and flavor compounds is responsible for the loss of flavor and shortened shelf life of foods (42). Only the aroma that interacts with the protein through non-covalent forces is conducive to the flavor characteristics of protein-containing foods. The interaction of flavor compounds with proteins is usually completely reversible, but in some cases the volatile flavor substances are covalently bound to proteins, which is usually irreversible. For example, the amino group of aldehydes and lysine residues, and the amino group and carboxyl group are irreversible. In meat and meat products, hydrophobic interaction is the main force to maintain the interaction between proteins and flavor substances, which is also a manifestation of the special folding of polypeptide chains and the tendency of protein structure to stabilize (43). In liquid and high-moisture foods, the mechanism of binding flavor substances to proteins mainly involves the interaction of non-polar flavor compounds with hydrophobic regions or cavities on the protein surface. In general, proteins with strong surface hydrophobicity will also directly adsorb flavor compounds on the molecular surface (44). For those protein molecules with weak surface hydrophobicity, flavor substances enter the protein molecules and bind to the hydrophobic point of the cavity (44). In addition, flavor compounds containing polar groups, such as hydroxyl and carboxylic groups, can combine with proteins through hydrogen bonding and ionic effects. In general, most interactions between proteins and flavor compounds are reversible, such as ionic bonding, hydrogen bonding, and hydrophobic interactions. Guichard (4) found that the hydrophobicity of proteins and flavor compounds showed a positive correlation during flavor-protein interactions (see Table 2).

**Table 2:** Method for analysis and detection of binding sites and binding constants of protein and flavor substances.

Method	Theory	Specific applications	References
Equilibrium dialysis	According to the principle of distribution balance of two different solutions, the content of flavor substances that were not combined with protein in equilibrium state was determined	Reference method for determining the number of proteins bound to small molecules	(33, 45)
Microdialysis	The molecular mass is controlled by trapping molecules of specific molecular mass through semi-permeable membranes	It has become one of the most important research tools in experimental neurophysiology and neurochemistry	(46, 47)
Affinity chromatography	Using protein as stationary phase, the whole affinity of protein can be reflected by measuring the retention time of flavor substance and calculating the binding constant	It is often used for biomolecular separation	(48–50)
Gas chromatography-mass spectrometry (GC-MS)	Various flavor compounds were separated from each other by GC column and entered the detector to be detected and recorded, and then the compound content was determined by MS analysis	The most commonly chosen method for detecting organic matter	(51, 52)
Differential scanning calorimetry (DSC)	The energy required to increase the temperature of the solution is measured to obtain information about structural changes during heat-induced protein unfolding	It is used to study the structure and function of biofilms and the conformational changes of proteins and nucleic acids	(53–55)
Isothermal titration	Directly measure the amount of heat released or absorbed during molecular bonding	It is one of the classical methods for detecting and characterizing biomolecules	(56–58)
Circular dichroism spectroscopy	On the basis of protein conformation changes, the changes of protein conformation before and after binding small molecules were characterized	The spatial structure of DNA and proteins can be determined	(59, 60)
Fluorescent spectrometry	The fluorescent donor and acceptor were labeled on the protein molecule, and the fluorescence intensity and the simple resonance energy transfer efficiency between the donor and acceptor were quantitatively determined. Finally, the distance between the labeled sites at the fitting site was determined	The main means to study the interaction between small molecules and nucleic acids	(61, 62)
Liquid chromatography	The chemical shifts of each atom in the protein structure that can form NMR marks are obtained by isotopically labeled protein samples and processed by NMR technology	High boiling point, poor thermal stability, relative molecular weight (greater than 400 or more) of organic matter in principle can be used to separate, analysis by HPLC	(63–65)

### 3.2 Method for analysis and detection of binding sites and binding constants of protein and flavor substances

The spatial conformation changes of proteins in meat products affect the binding degree of volatile flavor substances, and these changes can be detected by chromatography, mass spectrometry and spectroscopy. At present, many relevant studies focus on fluorescence spectroscopy, UV–VIS absorption spectroscopy, circular dichroism spectroscopy and molecular docking to analyze the mechanism of protein adsorption of volatile flavor substances. The following reviews the mechanism of protein adsorption of volatile flavor substances from three aspects: the degree of flavor adsorption, the change of protein structure, the force and the action site.

#### 3.2.1 Analysis of the degree of protein adsorption of volatile flavor substances

The degree of protein adsorption of volatile flavor substances is mainly resolved by liquid chromatography with gas chromatography-mass spectrometry (GC-MS) (27). The structural changes of proteins are mainly analyzed by thermodynamic methods and circular dichroism spectroscopy, with thermodynamic methods having the advantages of small sample size and high method sensitivity. The force and site of volatile flavor adsorption by proteins are mainly analyzed by spectroscopy, nuclear magnetic resonance (NMR) and molecular docking techniques. Currently, fluorescence spectroscopy, ultraviolet-visible absorption spectroscopy, circular dichroism spectroscopy and molecular docking are more frequently applied to analyze the mechanism of protein adsorption of volatile flavor substances.

#### 3.2.2 Protein structure analysis of adsorbed volatile flavor substances

The flavor substances such as aldehydes, alcohols, esters, sulfur-containing and nitrogen-containing have the characteristics of fat solubility and hydrophobicity (66). At present, omics methods are mainly used for analysis, including GC-MS, thin layer chromatography (TLC), matrix-assisted laser desorption ionization time-of-flight MS (MALDI-TOF-MS), electrospray ionization MS (ESI-MS) Q-Exactive high-resolution MS and NMR. Single technology cannot fully reflect the interaction between molecules, has certain limitations, can be used in combination with MS, multi-spectrum, molecular simulation and other technologies (67).

#### 3.2.3 Analysis of the forces and sites of adsorption of volatile flavor substances by proteins

Commonly used instrumental analysis methods to study the interaction between protein and flavor components include: gas chromatography-mass spectrometry, electronic nose (68); high performance liquid chromatography-mass spectrometry; gas chromatography-olfactory measurement (69, 70). Common detection methods: fluorescence spectrometry, differential scanning calorimetry, infrared spectroscopy, sedimentation velocity analysis, light scattering method, circular dichroic chromatography.

### 3.3 Factors affecting adsorption

When the flavor diffuses into the interior of the protein molecule, it destroys the hydrophobic interaction between the protein chain segments and destabilizes the protein structure, thus changing the protein conformation. For instance, flavor substances containing active groups, such as aldehydes, covalently binds lysine residues to promote the exposure of protein hydrophobic groups. As the protein molecule increases in size, it has a corresponding increase in the ability to absorb flavor substances, and the ability of proteins with porous properties to absorb flavor substances is significantly enhanced (71). After protein denaturation, the ability of proteins to absorb flavor substances increases after denaturation because more hydrophobic groups are exposed when the hydrophobic bonds that maintain the spatial helical structure of proteins are broken, enhancing the uptake of nonpolar flavor compounds. Any factor that can change the conformation of a protein can affect its binding to flavor substances, except for factors in the protein itself, such as moisture, temperature, pH, and the presence of salts and lipids. The interaction between muscle proteins and volatile compounds is related to protein properties, flavor types, ionic strength, pH value, protein oxidation and heating temperature, but there is no unified mechanism. Protein concentration, heating temperature, pH value and protein oxidation can induce the change of protein conformation, and then affect the interaction between protein and flavor substances (72). It is a complicated and arduous systematic project to study the adsorption properties of proteins for flavor. Therefore, it is of great significance to explore the factors that induce the change of muscle protein structure and how the structural changes affect the interaction between muscle protein and flavor substances.

#### 3.3.1 Protein

MP exhibits varying adsorption capacities for different flavor compounds, which is closely associated with the nature of flavor compounds (73). The adsorption binding of MP to flavor substances is altered by changes in protein concentration and properties. In food systems with high water content, the mechanism of protein interaction with flavor substances depends not only on water content, but also on the number and structure of protein side chains (74). As the concentration of volatile compounds increases, the more volatile compounds added to the water phase, the more volatile compounds F-actin binds (34). Compared with G-actin and actomyosin, F-actin binds to higher amounts of 3-methyl-butyraldehyde, 2-methyl-butyraldehyde, methoxy, caproaldehyde, and 2-pentanone. On the other hand, in the case of 3-methyl-butyral, caproaldehyde, methoxy, and octylaldehyde, the number of moles of G-actin binding is reduced and these volatile compounds are released into headspace. Under conditions of higher actomyosin and G-actin concentrations, octylaldehyde, 3-methylbutyral, hexal, methioaldehyde, and octylaldehyde are released into headspace, probably due to the presence of proteins that weaken their interactions (75). With the increase of protein concentration, the release of flavor components may be due to the enhanced interaction between proteins to weaken the adsorption between proteins and flavor components. Gu et al. (76) studied the interaction between carbonyl compounds and bovine serum protein, and also found that with the increase of bovine serum protein concentration, the interaction between proteins was strengthened, while the interaction between proteins and flavor distribution sites was weakened. In addition, it is also possible that the increase in protein concentration leads to a decrease in surface tension and thus an increase in the release of flavor components. O’Neill (5) also pointed out that MP is an effective surface tension inhibitor. Changes in protein conformation can also affect its effect on flavor substances, possibly due to changes in available protein binding sites that affect the interaction between volatile compounds and proteins (77).

#### 3.3.2 pH

Potential of hydrogen is one of the important factors that affect the binding ability of food protein and flavor compounds. Potential of hydrogen can change the secondary structure of proteins by changing the microenvironment of amino acid residues, surface hydrophobicity, protein aggregation degree, protein solubility, amount of charge carried by protein molecules, and inducing protein denaturation (78), thus changing the non-covalent interaction between protein molecules and flavor substances. Shimizu et al. (79) studied the structural characteristics of β-lactoglobulin at different potential of hydrogen, and the effect of β-lactoglobulin on flavor components was stronger at high potential of hydrogen. Gianelli et al. (80) reported the effect of curing agents treated with different potential of hydrogen on the binding ability of soluble protein and flavor compounds in skeletal muscle in the model system. It was confirmed by calculating the thermodynamic binding parameters (binding sites *n* and binding constant *K*) that potential of hydrogen treatment affects the ability of soluble proteins to bind flavor compounds in the model system. When Shen et al. (78) studied the mechanism of structural changes induced by different potential of hydrogen, they found that the adsorption capacity of MP to pyrazine compounds was jointly affected by pH value and pyrazine compounds, and the protein–protein interaction was enhanced at low potential of hydrogen (4.9–5.5), resulting in the formation of large-particle aggregates of MP. In addition, the binding fluorescence quenching and thermodynamic parameters were used to study the effect of pH-induced conformation changes on the interaction mechanism of MP with pyrazines. It was found that with the increase of potential of hydrogen (pH 5.0–8.0), the adsorption capacity of MP to aldehydes and esters increased, while the adsorption capacity of ketones decreased. The results showed that electrostatic and hydrophobic action were the main binding forces of MP and 2,5-dimethylpyrazine. The interaction is greatly influenced by potential of hydrogen, and at lower potential of hydrogen conditions (pH 4.9), protein–protein interactions are enhanced, MPs aggregate into larger particles, and surface hydrophobicity increases, thus exposing more hydrophobic binding sites (81). However, with the increase of surface hydrophobicity, the interaction between proteins (aggregation and sedimentation) is enhanced, and the steric hindrance generated prevents the binding of proteins with flavor substances, and the flavor release behavior of proteins is enhanced. In addition, establishing a correlation between pH-induced myofibrillar conformational changes and flavor substance interactions is critical for controlling meat-flavored myofibrillar-containing products.

#### 3.3.3 Temperature

Temperature mainly affects the flavor adsorption capacity and the number of action sites of proteins, in addition, it also affects the flavor release by affecting the rheological properties of food and the distribution coefficient of flavor components (82). The influence of temperature on adsorption is based on the change of protein structure, especially the thermal denaturation of protein, but different sites of action are affected differently by temperature (82, 83).

Heat treatment is one of the most important steps in food processing and is commonly used in meat processing (84). The effect of heat treatment on protein flavor adsorption capacity has also been studied for many times. During the process of protein structure development and polymerization, the flavor of meat products can be changed by modification of specific binding sites or exposure of more hydrophobic flavor binding sites. Kang et al. (85) found that the structure of beef myofibrillar protein would change to different degrees with the increase of temperature and the extension of heating time. Lv et al. (10) studied the effects of different temperatures (45, 55, 65, 75, and 85°C) on myosin structure and flavor adsorption capacity, they pointed out that the interaction of myosin with aldehydes at different heating temperatures mainly depended on hydrophobicity, and that sulfhydryl and hydrogen bonding interactions determined the unfolding and polymerization of myosin structure, providing or modifying more flavor-binding sites, which in turn affected the adsorption of aldehydes. Zhou et al. (86) found that the secondary structure and surface hydrophobicity of myofibrillar gel changed within 0–5 min of heat treatment, which was consistent with the change time of the adsorption capacity of myofibrillar gel for different volatile flavor substances affected by heat treatment. The results showed that the changes of protein structure were related to the adsorption of volatile flavor compounds. Xu et al. (87) studied the effects of two-step heat treatment on the structure of myofibrillar protein of grass carp and its binding ability with hexal, heptyl, caprylic and nonaldehyde. The results showed that the surface hydrophobicity and total sulfhydryl content of myofibrillar protein increased within 30 min of the first heating at 40°C and 5–10 min of the second heating at 90°C, respectively. It may be due to the exposure of hydrophobic amino acids and sulfhydryl groups that lead to the unfolding of the secondary structure of myofibrillar protein, thereby improving the flavor binding capacity of myofibrillar protein. Prolonged heating at 90°C accelerates the aggregation of unfolded myofibrillar protein, reduces the hydrophobic binding site, and thus reduces the binding capacity of myofibrillar protein to aldehydes.

In addition to protein, animal meat also contains reducing substances such as ribose and glucose, which underwent maillard reactions during heat treatment, not only giving meat products their characteristic flavor and color, but also affecting the digestion and utilization of protein. Setyabrata et al. (88) compared the volatile flavor components of yak meat myofibrillar protein with those of fructose and glucose in the melad reaction. At a reaction temperature of 130°C, fructose and glucose were involved in the melad reaction to produce the most abundant and the highest content of volatile flavor components, and the types and contents of flavor components detected in the melad reaction involving glucose were significantly lower than those of fructose.

In addition to heat treatment, low temperatures also affect the interaction of myofibrillar proteins with flavor substances. The presence of fresh myosin resulted in a significant decrease (*p* < 0.05) in the percentage of free hexanal, methylthioal, and octanal, which indicated the ability of fresh myosin to bind these volatile compounds. However, the effect of frozen storage on actomyosin binding ability was different. The percentage of free caproaldehyde and methioaldehyde increased significantly when actomyosin was frozen with or without glycerin. This means that less volatile compounds are bound compared to fresh actomyosin. When actomyosin was frozen with glycerin, the percentage of free octaldehyde decreased significantly, while the percentage of free octaldehyde increased without glycerin. Fresh actomyosin is not bound to 3-methylbutyraldehyde, 2-methylbutyraldehyde and 2-pentanone, and the dissociation rate of these compounds is not affected by freezing when glycerol is used, but the dissociation rate of these compounds is significantly reduced when glycerol is not used (89).

#### 3.3.4 Flavor substance

The binding of MP to flavor substances is also affected by the types and properties of flavor substances. Hexal, heptyl, octyl, nonylal and 1-octene-3-ol are the main volatile substances that provide meat flavor (90) (E)-2-decenal (E, Z)-2,4-decenal, and (E, E)-2,4-nonadienal have a strong meat flavor, and dimethyl trisulfide and phenylacetaldehyde contribute to the formation of meat flavor (84). These 10 volatile substances have important effects on the formation of meat flavor.

In terms of flavor substance types, it was found that MPs adsorb several typical flavor compounds such as alcohols, aldehydes, ketones, and esters with different strengths and effects. The adsorption of aldehydes, aldehydes, ketones and esters by the same concentration of protein was in the order of aldehydes > esters > ketones > alcohols from strong to weak. Myofibrillar protein has little adsorption effect on lower alcohols. This is similar to the findings of Kühn et al. (91), possibly because there is no significant interaction between the hydroxyl group of alcohols and the amino group of proteins. However, with the increase of carbon chain length, the hydrophobicity of alcohols increases gradually, and myofibrillar protein adsorbs them to some extent through hydrophobic interaction. In addition, among various flavor substances, ketones are the main compounds in the flavor of meat products, contributing significantly (92).

The difference of molecular structure of flavor compounds can also lead to the difference of adsorption capacity of MP to flavor compounds. For similar flavor compounds, the larger the molecular weight, the longer the carbon chain, the stronger the molecular hydrophobicity, the stronger the hydrophobic binding ability of proteins, and the higher the adsorption efficiency (93). On the contrary, the larger the molecular volume of the compound, the more obvious the steric hindrance effect, the more difficult it is to enter the binding site inside the protein molecule, resulting in low adsorption efficiency (94). Hansen et al. (95) found that the adsorption capacity of proteins to ketones increased with the increase of the number of carbon atoms. Han et al. (96) found that the interaction between nonylaldehyde and MP had no significant effect on its conformation, and the binding constant and number of binding sites increased with the increase of temperature.

Another important factor that plays a key role in food flavor binding is the stability and reactivity of flavor substances. Weerawatanakorn et al. (97) conducted a comprehensive review of the chemical reactivity of 10 food flavor compounds frequently detected in food. In general, active functional groups, such as hydroxyl, sulfhydryl and carbonyl groups, can affect the chemical reactivity of these compounds, or can affect the physical or chemical interactions between flavor agents and various MPs. During food processing, including heat treatment, flavor compounds not only interact with proteins, but also easily undergo structural changes through various chemical reactions. Such as the degradation of free amino acids, the oxidation of aldehydes to acids, and the rearrangement and isomerization of terpenes under acidic conditions (98). The “kinetic” reactions of flavor substances can affect the overall flavor characteristics of foods, making the study of protein-flavor interactions more complicated (99). Due to the complexity of the food system, flavor substances can simultaneously interact with other components in the food matrix such as water, lipids, carbohydrates, vitamins, and minerals. Nevertheless, for protein-rich foods, such as dairy and meat products, protein is still considered to be an important component that causes flavor loss or release (8).

#### 3.3.5 Other external factors

The interaction between proteins and flavors is not only affected by intrinsic factors such as the properties of proteins and flavors, as well as pH, temperature, protein hydration capacity, but also by other external factors such as ionic strength and oxidation conditions (100). In addition to the internal mechanism of binding, factors such as enzymatic reactions, high pressure, microwaves, or pulsed electric fields can also be affected, and they are the basic methods for altering protein-flavor interactions by changing the external or internal environmental conditions of the food system (101). These factors can influence the binding behavior of proteins and flavors by changing the structure of proteins，and they are reflected by binding parameters.

The structure of MP and the sequence of adsorption of flavor substances. Due to the differences in the chemical structure and functional groups of proteins and flavor compounds, there is currently no general theory to explain protein-flavor substance interactions (102). In general, studies tend to consider the binding strength of flavor components to proteins as follows: aldehydes are greater than ketones and alcohols are greater than alcohols (91, 102). When the flavor molecules have the same chemical identity, high flavor retention effects can be observed as the length of the fat chain increases (103). Ma et al. (104) studied the interaction of pyrazine compounds 2-methylpyrazine (MP), 2,5-dimethylpyrazine (DP), 2,3,5-trimethylpyrazine (TRP) and 2,3,5,6-tetramethylpyrazine (TEP) with proteins. To elucidate the effect of alkyl distribution in pyrazine ring on flavor release in bovine serum albumin (BSA) solution (pH 7.0). The results of SPME-GC-MS showed that the distribution of methyl group in pyrazine ring significantly affected its release from BSA solution. The release sequence of pyrazine compounds in BSA solution was MP > DP > TRP > TEP. Yin et al. (105) studied the interaction between furan derivatives and pork MPs and observed that the binding capacity of MPs decreased in the order of 5-methyl furfural > furfural >2-acetyl furan > furanol. The results indicate that hydrogen bonding, van der Waals forces, and hydrophobic interactions are the main ways that MPs interact with the four furan derivatives. Another study conducted by Menezes et al. (106), who confirmed that carboxyl derivatives of furfural have a higher affinity for BSA and human transferrin than ketone derivatives. The additional H-bond between the carboxyl derivatives of furfural and the protein contributes to the most stable complex. These results suggest that both the structure of flavor homologues and the properties of proteins in the food substrate can influence the release or retention of aroma compounds. The adsorption of proteins on certain and similar flavor substances also needs to be studied according to specific conditions to determine the influence of differences in physical and chemical properties of flavor substances on protein adsorption (15, 74, 93, 103, 107). In general, the adsorption efficiency of protein for aldehydes is higher than that for esters, ketones and alcohols, indicating that functional group activity and location play an important role in the protein-flavor compound interaction. In addition, hydrophobicity and steric hindrance of flavor compounds also play an important role in protein adsorption of flavor molecules. For similar flavor compounds, the larger the molecular weight and the longer the carbon chain, the stronger the molecular hydrophobicity, the stronger the protein’s hydrophobic binding ability, and the higher the adsorption efficiency. At the same time, the larger the molecular volume of the compound, the more obvious the steric hindrance effect, the more difficult it is to enter the binding site inside the protein molecule, resulting in low adsorption efficiency. Since the binding of flavor compounds to proteins is mainly attributed to the active groups of flavor molecules, steric hindrance effects are also mainly reflected in the effects on functional groups. Therefore, the adsorption effect of protein on flavor compounds should be considered by various factors, including not only the change of protein structure, but also the physicochemical properties of flavor compounds.

Scatchard (or Klotz) Since most protein-flavorant interactions can be attributed to reversible non-covalent binding, the classical theoretical models used to describe flavorant compounds in equilibrium with proteins follow the Scatchard (or Klotz) equation (108) Eq. 1 or Hill or the Hill model (109) Eq. 2:


(1) $$\frac{1}{v}=\frac{1}{n}+\frac{1}{nK\left[L\right]}$$

(2) $$\frac{1}{v}=\frac{1}{n}+\frac{1}{n{\left(K\left[L\right]\right)}^{h}}\;$$

where *v* is the number of moles of flavor ligand bound by protein per mole; n is the number of binding sites on the protein; *K* and [*L*] are the binding constant (mol/L)^−1^ and the concentration of the free flavoring ligand in solution at equilibrium (mol/L). The binding constant *K* can be thought of as the gas–liquid partition coefficient, which defines the concentration ratio of the flavor agent in the air and liquid phase at equilibrium. In general, the binding of flavor and protein is a spontaneous process, and the binding mechanism can be divided into two groups from the perspective of thermodynamic parameters: driven by enthalpy or entropy (110, 111). The enthalpy-driven process shows that the binding of flavors and proteins can occur at low temperatures, and that the forces of interaction between them include van der Waals forces and hydrogen bonds. In addition, the binding of flavor to protein is entropy-driven, which means that high temperatures favor the interaction. In other words, heat treatment or other processes that can induce protein unfolding are more conducive to promoting flavor-protein interactions (112–114). Previous studies have evidenced that modification of protein conformation exposes more hydrophobic interaction sites, which may contribute to the binding of flavor proteins (115).

In the post-2018 study, additional mathematical models were developed to predict flavor distribution in protein solutions. Tan et al. (116) constructed the equation model by partial least squares regression (PLSR) as a function of molecular descriptors describing the binding behavior of BSA and four types of flavor ligands (ketones, esters, aldehydes and alcohols) in the aqueous state. Viry et al. (43) attempted to evaluate the mechanism of protein-flavor binding using a practical dairy system containing caseinate and whey protein, and proposed a mathematical model for flavor distribution in protein solutions. It is assumed that the interaction between the flavor agent (F, 0.05–0.7 mg/kg) and the protein (P, 3–9%, w/v) is limited to bimolecular interactions and that no other interactions are involved in the reaction system. The result of appeal showed that the hydrophobicity of the flavor played a leading role in the retention of esters, alcohols and other flavor substances. Recent studies have confirmed that MP can bind to flavor substances such as ketones, alcohols, esters, aldehydes and some sulfur-containing compounds, which affects the final quality of meat products by changing the functional properties of proteins (15, 117). Lou et al. (103) found that the binding ability of ketones (2-heptanone, 2-pentanone, 2-nononone and 2-octanone) was positively correlated with the hydrophobicity and sulfhydryl content of G-actin in carp. The unfolding of G-actin secondary structure may expose new flavor compound binding sites, while the refolding and aggregation of proteins may be responsible for the decreased binding ability (87). To further elucidate the influence of ketone molecular structure, a study conducted by Shen et al. (118) systematically evaluated the binding behavior of MP to ketone flavor. By comparing the binding capacity of MP with 5-methyl-2-hexanone, 2-nonone, 2-pentanone, 2-heptanone, 2-octanone, 3-pentanone, 2,3-pentenedione and 2-nonanone, the results showed that the number and location of keto groups, molecular polarity and size played an important role in the interaction mechanism. The properties of MP have an insignificant effect on flavor binding behavior. In addition, Pérez-Juan et al. (15) found that MPs do not seem to be primarily responsible for volatile compound interactions, while sarcoplasmic proteins are important. The conformation and concentration of myofibrillar protein determines the binding capacity of the protein, and while F-actin can bind higher amounts of flavor compounds, G-actin cannot bind any selected flavor compounds.

In addition, computational methods for studying proteins have become increasingly powerful and popular in recent decades. Molecular docking and MD simulation are powerful tools for studying protein-ligand interactions, which can be used to aid research. Molecular docking is often used to find potential high-affinity ligands from a large number of different chemical libraries and to predict possible binding patterns. Molecular docking programs are commonly used in drug development and are favored because of their low computational cost. MD requires more computational resources than molecular docking, but allows research to study the dynamic properties of proteins. MD simulation has contributed to the study of protein folding, drug design and protein engineering. While molecular docking is commonly used for drug discovery, it has many other applications, such as protein engineering. Protein engineering is used to modify existing proteins to increase stability or change function, or to design entirely new protein sequences. Typically, the goal of protein engineering is to interact with modifications of small ligands. In this case, the binding of molecular docking with MD is an ideal way to characterize this interaction. Molecular docking can predict the binding posture and binding affinity of ligands, while MD adds flexibility, solvation effects, and dynamical information about bonds. Although both molecular docking methods and MD methods are useful individually, they complement each other and together facilitate many studies (119). Ren et al. (120) developed an enhanced sampling scheme for multi-domain proteins (generalized replica exchange with solute backfire selected surface-charged residues: gREST_SSCR) and applied it to ligand-mediated conformational changes in ribo-binding protein (RBPG134R) G134R mutants in solution.

## 4 Conclusion

In this paper, we reviewed the research progress of the interaction between MP protein and flavor substances in recent years, as well as the factors affecting the interaction and the methods of analysis and detection of binding sites and binding constants, which are helpful for the design and formulation of specific flavors in meat products, and also for qualitative and quantitative prediction of flavor binding behavior. In addition, we discuss in detail the influence of other components of the food system such as protein, moisture, temperature (thermal denaturation), pH, etc. on protein-flavor interactions.

At present, the adsorption of protein on flavor compounds is basically studied by constructing protein solution model and studying the interaction mechanism of the two in solution system. However, it is not the state of existence of meat proteins that is liquid in actual production. Many foods are solid or semi-solid phase, and are composed of several food components and flavor mixtures, which are much more complex than protein solution system. How to more truly analyze the mechanism of protein adsorption flavor substances in meat product matrix will be an important research content. These complex systems are important for predicting and controlling flavor release in real food systems. This paper presents a preliminary evaluation of the effect of temperature on protein flavor. In recent years, the application of non-thermal technology in the food industry has become increasingly extensive, and it has outstanding advantages in improving energy efficiency, improving product quality, and developing new products. Most studies show that compared with traditional treatment, some new non-thermal technologies such as supercritical fluid extraction, pulsed electromagnetic field, high-power ultrasonic wave, etc. are effective methods to improve the interaction between proteins and flavor substances. These techniques have broad application prospects in the modification of protein flavor in the future. In addition, the method of analysis and detection of binding sites and binding constants is discussed in this paper. It is found that the verification of binding sites for protein adsorption of flavor substances and the calculation of force-determined flavor retention coefficient still need theoretical breakthroughs. How the modification of flavor substances on meat substrates affects the adsorption of flavor substances needs to be systematically analyzed. Future research directions may also be highlighted. Food matrix is a complex system, based on the study of a single protein, the study of complex protein system and multi-component food matrix and flavor components is also a future development trend.

## Author contributions

RQ: Writing – original draft, Writing – review & editing. CS: Writing – original draft, Writing – review & editing. TB: Formal analysis, Project administration, Writing – review & editing. JY: Funding acquisition, Writing – review & editing. JC: Formal analysis, Writing – review & editing. JZ: Funding acquisition, Project administration, Writing – review & editing.
